# Licochalcone A: A Potential Multitarget Drug for Alzheimer’s Disease Treatment

**DOI:** 10.3390/ijms241814177

**Published:** 2023-09-16

**Authors:** Jordi Olloquequi, Miren Ettcheto, Amanda Cano, Ana Fortuna, Joana Bicker, Elena Sánchez-Lopez, Cristian Paz, Jesús Ureña, Ester Verdaguer, Carme Auladell, Antoni Camins

**Affiliations:** 1 Departament of Biochemistry and Physiology, Physiology Section, Faculty of Pharmacy and Food Science, Universitat de Barcelona, Av. Joan XXIII 27/31, 08028 Barcelona, Spain; 2 Laboratory of Cellular and Molecular Pathology, Instituto de Ciencias Biomédicas, Facultad de Ciencias de la Salud, Universidad Autónoma de Chile, Talca 3460000, Chile; 3 Departament of Pharmacology, Toxicology and Therapeutic Chemistry, Faculty of Pharmacy and Food Science, Universitat de Barcelona, 08028 Barcelona, Spain; mirenettcheto@ub.edu (M.E.); camins@ub.edu (A.C.); 4 Biomedical Research Networking Center in Neurodegenerative Diseases (CIBERNED), 28031 Madrid, Spain; acano@fundacioace.org (A.C.); esanchezlopez@ub.edu (E.S.-L.); jurena@ub.edu (J.U.); everdaguer@ub.edu (E.V.); cauladell@ub.edu (C.A.); 5 Institute of Neuroscience, Universitat de Barcelona, 08028 Barcelona, Spain; 6 Institut d’Investigació Sanitària Pere Virgili (IISPV), 43005 Reus, Spain; 7 Ace Alzheimer Center Barcelona, International University of Catalunya (UIC), 08028 Barcelona, Spain; 8 Institute of Nanoscience and Nanotechnology (IN2UB), 08028 Barcelona, Spain; 9 Department of Pharmacy, Pharmaceutical Technology and Physical Chemistry, Faculty of Pharmacy and Food Science, Universitat de Barcelona, 08028 Barcelona, Spain; 10 Faculty of Pharmacy, University of Coimbra, 3000-548 Coimbra, Portugal; anacfortuna@gmail.com (A.F.); joana.bicker@gmail.com (J.B.); 11 Coimbra Institute for Biomedical Imaging and Translational Research (CIBIT), 3000-548 Coimbra, Portugal; 12 Unit of Synthesis and Biomedical Applications of Peptides, IQAC-CSIC, 08034 Barcelona, Spain; 13 Laboratory of Natural Products & Drug Discovery, Center CEBIM, Department of Basic Sciences, Faculty of Medicine, Universidad de La Frontera, Temuco 4780000, Chile; cristian.paz@ufrontera.cl; 14 Department of Cellular Biology, Physiology and Immunology, Faculty of Biology, Universitat de Barcelona, 08028 Barcelona, Spain

**Keywords:** neurodegeneration, cognitive enhancement, multi-target therapy

## Abstract

Licochalcone A (Lico-A) is a flavonoid compound derived from the root of the Glycyrrhiza species, a plant commonly used in traditional Chinese medicine. While the Glycyrrhiza species has shown promise in treating various diseases such as cancer, obesity, and skin diseases due to its active compounds, the investigation of Licochalcone A’s effects on the central nervous system and its potential application in Alzheimer’s disease (AD) treatment have garnered significant interest. Studies have reported the neuroprotective effects of Lico-A, suggesting its potential as a multitarget compound. Lico-A acts as a PTP1B inhibitor, enhancing cognitive activity through the BDNF-TrkB pathway and exhibiting inhibitory effects on microglia activation, which enables mitigation of neuroinflammation. Moreover, Lico-A inhibits c-Jun N-terminal kinase 1, a key enzyme involved in tau phosphorylation, and modulates the brain insulin receptor, which plays a role in cognitive processes. Lico-A also acts as an acetylcholinesterase inhibitor, leading to increased levels of the neurotransmitter acetylcholine (Ach) in the brain. This mechanism enhances cognitive capacity in individuals with AD. Finally, Lico-A has shown the ability to reduce amyloid plaques, a hallmark of AD, and exhibits antioxidant properties by activating the nuclear factor erythroid 2-related factor 2 (Nrf2), a key regulator of antioxidant defense mechanisms. In the present review, we discuss the available findings analyzing the potential of Lico-A as a neuroprotective agent. Continued research on Lico-A holds promise for the development of novel treatments for cognitive disorders and neurodegenerative diseases, including AD. Further investigations into its multitarget action and elucidation of underlying mechanisms will contribute to our understanding of its therapeutic potential.

## 1. Introduction

Alzheimer’s disease (AD) is a progressive neurodegenerative disorder and the most common cause of dementia worldwide [1]. The prevalence of this pathology continues to increase, reaching a current estimate of 22% among individuals aged 50 and above who experience some stage of the disease [2].

The key symptoms of AD initially include mild cognitive impairment and subtle memory loss of recent events, which progressively worsens to severe cognitive impairment with profound amnesia. In addition to memory loss, individuals may experience personality, behavioral, and motor changes [3]. AD is characterized by a lengthy asymptomatic stage of approximately 20 years, during which individuals do not exhibit cognitive impairments. However, even though the patient remains symptom-free, the disease has already commenced at a pathophysiological level. The disease ultimately leads to death, usually occurring 4–8 years after the diagnosis [3].

Early diagnosis plays a critical role in determining the efficacy of a treatment. Diagnosing AD involves clinical assessment and informant interviews, along with tests of memory and thinking skills. Blood and imaging tests can help rule out other potential causes of the symptoms and aid in identifying the disease-causing dementia symptoms. Biomarker tests, including specific types of PET scans and tests that measure amyloid and tau proteins, can detect the presence of plaques and tangles associated with AD [4]. These biomarkers provide a means for diagnosing the pathology during life with more certainty.

Currently, there is no cure or disease-modifying treatment for AD. However, treatment is available to manage the symptoms and improve the quality of life for affected individuals. Symptomatic therapy is the primary approach, focusing on controlling cognitive and behavioral symptoms [5]. However, these therapies lose efficacy as the disease progresses. There are also ongoing clinical trials and research efforts aimed at developing disease-modifying treatments and potential future therapies [6]. Currently approved pharmacological treatments for AD include cholinesterase inhibitors such as donepezil, rivastigmine, and galantamine. These aim to improve cognitive function by increasing acetylcholine levels but have modest efficacy and side effects like nausea, vomiting, and muscle cramps [7,8]. The N-methyl-D-aspartate (NMDA) receptor antagonist memantine is also used to reduce glutamate excitotoxicity, but evidence for its benefits in mild AD is insufficient [9,10]. These drugs only provide symptomatic relief and do not significantly slow disease progression. On the other hand, the monoclonal antibody Aducanumab initially did not succeed in a phase 3 trial that was randomized, double-blind, and placebo-controlled. However, a subsequent phase 3 study demonstrated its potential to decrease amyloid plaque levels, ultimately leading to the drug’s authorization [11]. Nevertheless, its efficacy is still controversial [12].

Licochalcone A (Lico-A) is a phenolic compound derived from the root extract of the Glycyrrhiza species that has demonstrated promising neuroprotective properties and therapeutic potential for AD [13]. Preclinical studies have reported the ability of Lico-A to reduce amyloid plaques and tau phosphorylation, exhibit anti-inflammatory and antioxidant effects, and inhibit acetylcholinesterase activity [14,15,16]. These multitarget actions suggest that Lico-A could have beneficial effects on key mechanisms implicated in AD pathogenesis. While research on Lico-A for AD treatment is still in the early stages, its demonstrated bioactivities make it an interesting natural compound to explore for cognitive enhancement and neuroprotection. In this review, we will discuss the existing evidence for Lico-A as a potential therapy for AD and related neurodegenerative conditions.

## 2. Pathophysiological Hallmarks of AD: Therapeutic Implications

The pathophysiological mechanisms of AD involve various processes that contribute to the progressive degeneration of the brain. Although the exact molecular mechanisms responsible for AD are still not fully understood, it is accepted that a combination of genetic, environmental, and lifestyle factors contributes to its development [17].

As in other neurodegenerative diseases, AD is characterized by the accumulation of toxic protein aggregates, including beta-amyloid (βA) plaques and neurofibrillary tangles (NFTs). βA plaques are abnormal deposits of a protein called βA peptide that accumulates between neurons. These plaques disrupt normal cell signaling and promote neuronal damage and cell death [18]. βA is derived from the breakdown of a larger protein called amyloid precursor protein (APP). In AD, there is an imbalance in the production and clearance of βA, leading to its accumulation and subsequent plaque formation [19]. NFTs are another hallmark of AD and are formed by the aggregation of a protein called tau. Tau normally helps stabilize microtubules, which are essential for maintaining the structure and transport within neurons. However, in AD, tau proteins become hyperphosphorylated, causing them to form tangles [20]. These tangles disrupt the normal functioning of neurons, leading to their degeneration and death [21].

Researchers have identified several genetic variants that play a role in AD. They are known as risk genes and deterministic genes. Risk genes, such as the apolipoprotein E (*APOE*) isoform e4, increase the likelihood of developing the disease but do not guarantee it [22]. Deterministic genes, on the other hand, have a stronger link to the development of AD, but they are rare. Autosomal dominant mutations on deterministic genes are associated with familial AD (FAD), a form of the disease that has a strong genetic component. Hence, there are rare mutations that have been identified in the precursor of βA, specifically in the C99 fragment of the APP, or in the protease responsible for its production, known as Presenilin (PSEN)/γ-secretase [23].

It has been hypothesized that the primary neuropathological markers, βA and NFTs, may initiate the neurodegenerative process that leads to cortical and hippocampal atrophy. Alongside βA and tau, multiple pathophysiological processes occur simultaneously, contributing to the progression of the disease. These pathological changes include synaptic dysfunction and/or loss, dysregulation of neurotransmitter systems (such as cholinergic and glutamatergic deficits [24,25]), vascular dysfunction [26], oxidative stress [27], neuroinflammation [28], and metal dysregulation [29]. Understanding these pathophysiological mechanisms is crucial for the development of effective diagnostic tools and therapeutic strategies to combat this devastating disease.

Although two decades of research have revealed many layers of complexity of AD pathogenesis, a bulk of data still supports a role for βA as the primary initiator of AD [30] and synapses represent the scenario in which βA exerts its initial toxic effects [31]. The hypothesis seems to be supported by two genetic findings: (1) genetic forms of AD produce an increase in βA or βA fibrillogenic properties and are sufficient to induce typical AD pathology [32]; (2) APOE e4 appears to increase AD risk by affecting βA seeding and clearance [33,34]. However, even though the genetic evidence strongly supports the relevance of βA aggregation in triggering the AD cascade, it seems clear that βA is necessary but not sufficient and that other downstream factors play a key role. For example, the correlation between βA deposition and cognitive decline is minimal [35] and regional cerebral βA deposition does not correlate with patterns of regional cerebral hypometabolism detected via functional neuroimaging [36]. Furthermore, in the last few years, the amyloid hypothesis has faced skepticism due to the ineffectiveness of βA-based therapeutics that appear to be ineffective in modifying the disease course for symptomatic AD [37]. Indeed, the monoclonal antibody against βA aducanumab, which was conditionally approved in the USA, was recently withdrawn. Moreover, in January 2023, the U.S. FDA approved Lecanemab, an anti-amyloid monoclonal antibody used for early-stage AD treatment. Despite this, there is currently no evidence to suggest that it can completely modify the progression of AD [38].

Nevertheless, it is conceivable that the sole blocking of the amyloidogenic pathway may not be appropriate given that the highly complex pathogenesis of AD is extremely complicated. Therefore, this simple straightforward pathway that depicts AD pathogenesis as a cascade of events triggered by βA deposition has been revised and deeply debated over the years [37,39,40]. In addition, numerous reports demonstrate that the loss of synaptic markers and/or dendritic spine precede the formation of βA plaques and NFTs, implying a strong correlation among these molecules and structures with cognitive impairment in AD [41,42,43]. Moreover, post-mortem analyses support the central role of synaptic loss in AD pathogenesis: quantitative correlations of AD cytopathology with cognitive deficits indicate that synapse loss is more robustly correlated than the numbers of βA plaques or NFTs, the degree of neuronal perikaryal loss, or the extent of cortical gliosis [44]. Overall, several biochemical and morphologic indicators suggest that AD represents an assault on synapses in the early stages of the disease [45,46]. In this sense, an update of the amyloid cascade hypothesis proposes a mechanism for memory loss based on the impact of small, soluble Aβ oligomers on synaptic function [39]. According to this hypothesis, early memory loss would result from synapse failure before neuron death, and Aβ oligomers, rather than fibrils, would trigger synaptic dysfunction. What seems clear is that the high concentration of βA in and around plaques causes localized damage to synapses [47], disrupting local networks [48].

Undoubtedly, aging is also a significant risk factor, with the incidence of AD rising sharply after the age of 65. However, in the asymptomatic stages, all the aforementioned pathophysiological events gradually accumulate until clinical symptoms manifest. For this reason, it is necessary to search for markers of the disease to detect it in these asymptomatic stages since identification of the prodromal phase of AD could be crucial for its treatment. Beyond the challenge of being able to detect the disease before it manifests, having an appropriate and effective therapy remains an unresolved issue in AD research. In this sense, although various drugs have been developed with diverse and attractive approaches for the treatment of AD, they were directed at a single objective or target and did not achieve the expected curative effect. Hence, monotherapies have failed to stop or delay the disease so far, and only showed modest symptomatic benefits [49,50].

Given the multifactorial nature of AD, multi-target therapies are increasingly demonstrating promising advantages. Multi-targeted ligands (MTDLs), which are designed using a combination of structurally active pharmacophores, are becoming attractive strategies for AD therapy. Hence, drugs simultaneously acting not only on the amyloidogenic pathway but on many other pathways can be a good strategy for the therapeutic dilemma of AD. For example, a series of tacrine derivatives have been developed to target various key features, including βA, tau protein, N-methyl-D-aspartate receptor, cholinesterase, monoamine oxidases, and secretases, with promising preclinical results [51].

## 3. Licochalcone A: A Natural Compound with a Multitarget Side

The process of cognitive deterioration is closely related to and is a characteristic of human aging. Furthermore, aging is regulated by a variety of systems in the body, such as the nervous system, the immune system, and the metabolic system. Medicinal plants and their extracts have been widely utilized for centuries, as they hold great promise as a source for developing safe and effective agents to treat a wide range of diseases, including neurodegenerative conditions. Among these natural compounds, particular attention has been given to antioxidants or free radical scavengers due to their significant pharmacological potential [52].

**Figure 1:**
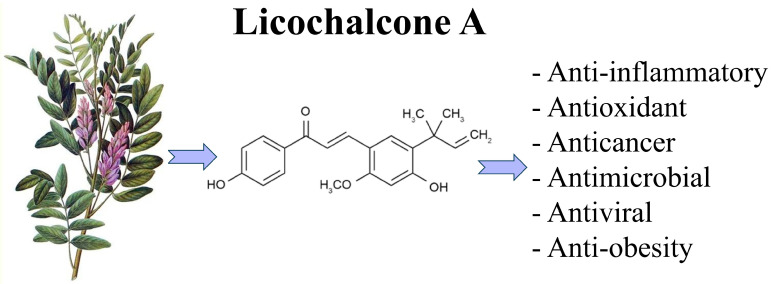
Pharmacological properties of Licochalcone A. Some studies have demonstrated various pharmacological bioactivities in Lico-A, including anti-inflammatory, antimicrobial, antiviral, antimycobacterial, and anticancer properties, indicating its potential therapeutic applications.

Licorice (Glycyrrhiza species), a Chinese herb listed in the Chinese Pharmacopoeia and the List of Herbal Materials, has been traditionally used for nutritional and medicinal purposes. Currently, licorice root remains one of the most prescribed herbs for the treatment of various diseases, such as microbial infection, inflammation, and cancer [53]. Licochalcone A is a type of flavonoid compound found in the root extract of licorice plants, with a characteristic chalcone structure (3-dimethylallyl-4,4′-dihydroxy-6-methoxychalcone). It is used as a food coloring and in the tobacco industry. Chemically, Lico-A is an α,β-unsaturated biphenolic ketone substituted with two phenolic hydroxyl groups, a methoxy moiety, and an isoprenoid side chain [54]. Lico-A has demonstrated various pharmacological bioactivities, including anti-inflammatory, antimicrobial, antiviral, antimycobacterial, and anticancer (Figure 1) [55]. Likewise, Lico-A seems to act in different mechanisms associated with the aging process. More specifically, Lico-A exhibits a diverse array of effects that target multiple aspects of AD pathology, including amyloid and tau accumulation, neuroinflammation, oxidative stress, and cognitive deficits.

**Figure 2:**
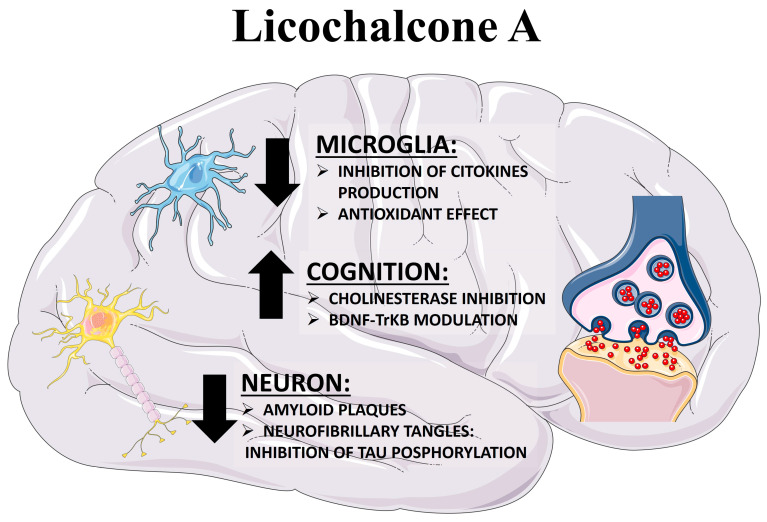
**Multitarget neuroprotective potential of Licochalcone A**. Lico-A has the potential to exert a neuroprotective effect through a variety of mechanisms. It not only enhances cognitive activity via the BDNF-TrkB pathway but also displays antioxidant and anti-inflammatory properties by inhibiting microglia activation, thereby mitigating neuroinflammation. Furthermore, as an acetylcholinesterase inhibitor, Lico-A contributes to elevated levels of the neurotransmitter acetylcholine in the brain. Additionally, it plays a role in reducing the presence of amyloid plaques and neurofibrillary tangles (NFTs), further demonstrating its multifaceted approach to neuroprotection.

In the next section, we will shift our focus to the pharmacological targets of Lico-A, which could potentially elucidate its effectiveness in enhancing cognitive processes during aging (Figure 2). We will discuss the key mechanisms of Lico-A that confer neuroprotection in AD, including PTP1B inhibition, anti-inflammatory effects, antioxidant properties, amyloid reduction, and acetylcholinesterase inhibition. Preclinical evidence on the neuroprotective effects of Lico-A is summarized in Table 1.

### 3.1. Licochalcone A against Diabesity-Associated Cognitive Loss

AD, obesity, and type 2 diabetes mellitus (T2DM) share several common risk factors, including advanced age, physical inactivity, hypertension, dyslipidemia, and genetic predisposition. By understanding these common mechanisms, we can better identify individuals who may be at a higher risk of developing ‘diabesity’, a condition combining T2DM with cognitive decline.

Insulin resistance, a key characteristic of T2DM, has been linked to impaired brain function and an increased risk of developing AD [65]. Indeed, insulin plays a crucial role in the brain, facilitating memory formation, synaptic plasticity, and neuronal survival [66]. Hence, it is not surprising that disruptions in brain insulin signaling pathways can lead to cognitive decline and neurodegeneration [67]. In this line, it has been shown that the physiological effects of insulin are primarily achieved through activation of the IRS-2/PI3K/AKT pathway. IRS-2 stands as one among several distinct types of insulin receptor (IR) substrate families. It exhibits abundant expression in the liver and plays a pivotal role in enhancing hepatic glycogen synthesis while inhibiting hepatic glucose production. In addition, IRS-2 serves as the central molecule for hepatic IR signal transduction [68]. A study by Luo and colleagues demonstrated that Lico-A effectively upregulates the expression of IRS-2, PI3K, and AKT in liver tissues, thereby amplifying the transduction effect on the PI3K/AKT signaling pathway involved in hepatic insulin regulation. These findings strongly suggest that Lico-A holds the potential to enhance insulin signal transduction, facilitating hepatic uptake and utilization of plasma glucose, and exhibiting promising anti-diabetic properties [69,70]. By improving insulin sensitivity and glucose metabolism, Lico-A emerges as a potential therapeutic agent for managing diabetes and its associated complications. In turn, Liou and colleagues conducted a study revealing the impact of Lico-A on weight gain in male C57BL/6 mice subjected to a high-fat diet (HFD) [71]. They observed that Lico-A effectively reduced body weight, along with a decrease in adipose tissue weight and adipocyte size in obese mice. Furthermore, Lico-A demonstrated its potential by improving blood glucose and insulin levels, resulting in a significant reduction in the HOMA-IR value, which signifies the amelioration of insulin resistance in obese mice. Moreover, at a preclinical level, Lico-A exhibited the ability to enhance the sirt1/AMPK pathway, thereby improving non-alcoholic fatty liver disease (NAFLD) [71]. Based on these findings, the authors concluded that Lico-A could serve as an anti-obesity agent for the treatment of NAFLD.

On another front, prior research has consistently indicated the significant involvement of c-Jun N-terminal kinases (JNK) in the progression of insulin resistance. Remarkably, studies using *Jnk1-/-* mice have demonstrated their notable protection against insulin resistance as well as their resistance to obesity induced by an HFD [72]. Moreover, in a study conducted by Zhu and colleagues, immunoblot analyses revealed a notable increase in phospho JNK levels in the brains of individuals with AD compared with control cases [73]. These findings suggest the existence of the dysregulation of JNK in the brains of AD patients, while the presence of obesity or T2DM could potentially contribute to neuronal stress in this context. One potential mechanism that could elucidate the relationship between JNK1-mediated brain insulin resistance and cognitive decline involves the phosphorylation of the insulin receptor substrate 1 (IRS1) on Ser-307. This phosphorylation event is believed to impede the activity of brain IR, leading to impaired insulin signaling [74,75,76]. Consequently, the JNK1-mediated phosphorylation of IRS1 emerges as a plausible direct cause of brain insulin resistance, which can contribute to cognitive impairments. Moreover, a study by Sze and collaborators showed that JNK1 activation increased tau phosphorylation in cultured neuroblastoma cells, while the JNK inhibitor SP600125 blocked tau phosphorylation and NFT formation [77]. Interestingly, Lico-A has been shown to be a JNK1 inhibitor [78], constituting a potential mechanism of action that provides favorable effects against cognitive decline associated with obesity and T2DM.

Finally, diabetic nephropathy, the most prevalent chronic microvascular complication of diabetes, has garnered significant attention. Preclinical studies have demonstrated the efficacy of Lico-A in effectively reducing blood glucose levels in mice subjected to an HFD [69]. Lico-A exhibits the capacity to regulate antioxidant enzymes like SOD and GSH-Px, as well as MDA, leading to notable enhancements in the renal oxidation index and mitigation of renal damage in mice afflicted with diabetic nephropathy [70]. These beneficial effects are attributed to the activation of Nrf2, which reduces oxidative stress damage.

All these results suggest that the antidiabetic effects of Lico-A could have the potential to reduce the cognitive decline associated with metabolic diseases.

### 3.2. Licochalcone A as a PTP1B Inhibitor

Protein kinases and phosphatases are families of enzymes that participate in the regulation of essential cellular functions through phosphorylation and dephosphorylation reactions. Among protein tyrosine phosphatases (PTPs), protein tyrosine phosphatase 1B (PTP1B) has received much attention due to its critical role in DMT2 and obesity since it acts as a negative regulator of the insulin and leptin signaling pathways [79].

Increased PTP1B activity is also associated with defective neuronal signaling of insulin and leptin [80,81], and these pathways are altered in AD [82,83]. It is known that hippocampal IR activation is key to ensuring good cognitive function in rodents [84]. In addition, Fuentes and colleagues have demonstrated that PTP1B shows a neuronal localization in dendritic spines and could be involved in the regulation of the cognitive process. In fact, the authors suggest that the lack of PTP1B promotes molecular and cellular conditions that may prime animals for enhanced learning [85]. Likewise, PTP1B negatively regulates the neuronal BDNF-TrkB pathway, involved in neuronal survival and synaptic plasticity, whereas PTP1B inhibition increases BDNF signaling [86,87]. PTP1B has also been shown to be involved in the hippocampal store-operated negative regulation of calcium influx (nSOC) [88], an essential process for spine stabilization [89,90]. Moreover, PTP1B is regulated by endoplasmic reticulum (ER) stress, which is also implicated in synapse loss and cognitive impairment in AD [91,92]. Furthermore, it has been shown that NMDA receptor signaling is affected by PTP1B in the hAPP-J20 murine model of AD. Thus, Zhang et al. (2021) discovered a significant impairment in long-term potentiation (LTP) of the CA3:CA1 synaptic response, which was effectively restored by either systemic inhibition of PTP1B or targeted elimination of PTP1B in glutamatergic neurons [93]. Finally, PTP1B is highly expressed in hippocampal microglia [94], being described as a positive regulator of neuroinflammation, which is also detrimental in AD [95]. Hence, it has been suggested that PTP1B regulates a variety of processes within the central nervous system (CNS), many of which are therapeutically relevant to AD [96].

Yoon and colleagues reported the inhibitory effect of the CH2Cl2 fraction extracted from *Glycyrrhiza inflata* on PTP1B [97,98]. In their research study, the investigators demonstrated that this extract contains various compounds, including Lico-A, which exhibited a significant inhibitory effect with an IC50 value of 19.1 µM ± 0.1. Furthermore, the same authors developed derivatives of Lico-A that showed even greater potency as PTP1B inhibitors [97]. Hence, PTP1B inhibition by Lico-A could be a promising target strategy to combat multiple cognitive and neurodegenerative aspects of AD, through modulation of different neuronal signaling pathways that enhance cognitive function and are affected in this disease, like BDNF/TrkB.

### 3.3. Licochalcone A as an Anti-Inflammatory Compound

It is widely known that the chronic neuroinflammatory process is an important feature of AD [99,100]. Microglia are the primary immune cells in the brain, and they seem to act as a double-edged sword in AD. On the one hand, they phagocytose and clear βA plaques, promoting neuronal survival [101]. On the other hand, prolonged microglial activation can lead to chronic neuroinflammation, exacerbating neuronal damage [102]. The activation of microglia in response to βA plaques is well established and βA oligomers have been shown to act by causing direct activation of microglia in primary microglial cultures [103]. In AD, microglia are often observed to be in an activated state, releasing proinflammatory cytokines, chemokines, and reactive oxygen species (ROS), contributing to neurotoxicity. Indeed, activated microglia are implicated in cognitive decline in AD through loss of synapses and the sustained secretion of neurotoxic cytokines, including TNF-α and IL-1β [104,105,106]. Specifically, TNF-α plays a pivotal role in cognitive decline by facilitating IRS1 phosphorylation through JNK activation, thereby modulating or inhibiting the IR in hippocampal neurons [82]. This process of TNF-α-induced neuronal insulin resistance has been proposed as a potential link between DMT2 and AD [107]. Similarly, IL-1β has been implicated in central inflammation-related cognitive impairment by altering synaptic plasticity in the hippocampus [108,109]. Moreover, activated microglia express iNOS (inducible nitric oxide synthase) producing nitric oxide (NO) that can cause a neurotoxic effect and also react with superoxide to produce peroxynitrite, which is also neurotoxic [110]. Apart from the neurotoxic effects triggered by neuroinflammation and oxidative stress, recent evidence suggests that the C1q/C3-CR3 signaling pathway promotes the elimination of synapses through microglial phagocytosis [111], representing a potential mechanism of cognitive function impairment in AD. Therefore, targeting microglial activation and the associated neuroinflammatory response has gained considerable attention in AD research as a potential disease-modifying treatment.

The activation of toll-like receptor-4 (TLR4) emerges as a crucial target in the neuroinflammatory process. TLR4 is expressed in astrocytes, microglia, and neurons, playing a significant role by recognizing both exogenous and endogenous ligands. This recognition triggers sustained neuroinflammation and neurotoxicity, resulting in cognitive impairment and an elevated risk of AD [112]. Notably, βA has been found to bind to TLR4 on the surface of microglia and astrocytes, initiating the release of proinflammatory factors. Activation of TLR4 subsequently triggers signaling pathways like MAPK (JNK, p38, and ERK) and NF-κB, culminating in the production of pro-inflammatory cytokines [113].

Lico-A has emerged as a promising candidate for modulating microglial function in AD and other neurodegenerative diseases (Figure 2). Some studies have unveiled the anti-inflammatory properties of Lico-A, which act by impeding the activation of MAPK and NF-κB, thereby suppressing the TLR4 signaling pathway [63]. Zhu and colleagues have proposed that Lico-A’s anti-inflammatory activity stems from its direct inhibition of Myeloid differentiation 2 (MD2), a TLR4 co-receptor involved in recognizing LPS. The interaction between LPS and TLR4/MD2 complex prompts downstream activation of intracellular signaling pathways (MAPKs and NF-κB) [114]. Moreover, it has also been reported that Lico-A can inhibit the production of NO, IL-6, and PGE2, hence, exerting neuroprotective and anti-inflammatory properties through iNOS target inhibitory activity [62]. Moreover, Li and colleagues reported that Lico-A suppressed p38 phosphorylation and the release of pro-inflammatory factors such as TFN-α, IL-1β, and IL-6 in animal models with chronic neuropathy [64]. In turn, in a preclinical model of Parkinson’s disease, systemic treatment with Lico-A significantly prevented the decline in dopaminergic neurons and inhibited the activation of microglia cells and the production of proinflammatory mediators [115]. Moreover, it has also been demonstrated that in the kainic acid animal model of epilepsy, Lico-A administration decreased neuroinflammation and attenuated the neurodegeneration process in hippocampal neurons [60].

From a different point of view, Wu and colleagues suggest that activating the immune system using Lico-A could enhance cognitive ability [116]. The authors reported that administration of Lico-A to rodents improves cognitive ability by inducing T cell proliferation in the spleen and whole blood.

In any case, whether by promoting peripheral activation of the immune system or exerting an anti-inflammatory action on microglia, Lico-A represents a potential therapy against AD and cognitive loss [117,118]. The anti-inflammatory properties of Lico-A could help mitigate neuroinflammation, a key process in AD progression involving activated microglia and the release of proinflammatory cytokines like TNF-α and IL-1β.

### 3.4. Licochalcone A as an Antioxidant Compound

As mentioned above, Nrf2 holds immense significance as an antioxidant sensor and serves as a key transcription factor essential for mitigating oxidative stress- and inflammation-related ailments [119]. In normal physiological conditions, Nrf2 remains confined to the cytoplasm, bound by its repressor protein, Kelch-like ECH-associated protein 1 (Keap1). However, when confronted with oxidative stress, cytosolic Nrf2 undergoes translocation into the nucleus where it binds to antioxidant response elements (AREs) present in the promoter regions of target genes. This binding event initiates the transcription process, leading to the subsequent expression of antioxidant enzymes like heme oxygenase-1 (HO-1), NAD(P)H quinone dehydrogenase 1 (NQO1), and glutamate-cysteine ligase (GCL). These enzymes assume a vital role in neutralizing ROS and upholding cellular redox homeostasis [120].

Lico-A has demonstrated antioxidative properties in L-02 cells, and studies have reported its ability to activate Nrf2-mediated antioxidant response signaling in RAW 264.7 cells exposed to oxidative stress [58,59]. The activation of Nrf2, along with its downstream genes such as HO-1, plays a protective role in combating cellular oxidant responses [121]. In rat primary cortical neurons, Lico-A treatment effectively counteracts the inhibition of the Nrf2 signaling pathway induced by oxygen–glucose deprivation/reoxygenation. Lico-A achieves this by enhancing the activity of SIRT1, a regulator of physiological functions associated with oxidative stress, as well as promoting Nrf2 activation [16]. Moreover, Lico-A is also involved in the activation of the AMPK/SIRT1 pathway [61]. Chen and colleagues demonstrated that Lico-A can dose-dependently reduce cellular oxidative stress in L-02 cells by increasing the activity of antioxidant enzymes such as SOD, CAT, and GPx [59]. These enzymes serve as the first line of defense within cells by catalyzing the inhibition of excessive oxidative stress.

Apart from its Nrf2 activation capabilities, Lico-A also demonstrates direct antioxidant activity by effectively scavenging free radicals including superoxide anions, hydroxyl radicals, and hydrogen peroxide [122]. Furthermore, Lico-A exhibits the capacity to chelate transition metals like iron and copper, which are recognized catalysts for the generation of highly reactive ROS [123]. This chelation action helps prevent the formation of damaging oxidative species. Additionally, Lico-A exerts an inhibitory effect on lipid peroxidation, which serves as a vital mechanism for halting the propagation of oxidative damage within cellular membranes [124].

Collectively, these results suggest that, in addition to its anti-inflammatory effects, Lico-A could confer neuroprotection in AD through its antioxidant actions, which could help counteract damaging oxidative stress, a key mechanism in AD’s pathogenesis.

### 3.5. Licochalcone A as Amyloid Inhibitor

As previously discussed, AD is characterized by the presence of βA plaques, which serve as histopathological markers. These plaques are formed in the brain due to the misfolding and self-association of amyloid protein, resulting in the formation of transient βA oligomers and fibrils. The misfolded βA peptides, comprising 39 to 43 amino acids, self-assemble to form βA protofibrils. Notably, the βA (1–42) protofibril, generated by the misfolding and aggregation of βA (1–42) fragments, is known to aggregate faster and possess increased neurotoxicity, playing a crucial role in the amyloid plaque formation process [125].

In a study conducted by Fang and colleagues, Lico-A’s destabilizing effects on the βA (1–42) protofibril are demonstrated. Molecular docking simulations revealed that Lico-A induces conformational alterations, leading to the destabilization of the βA protofibril (1–42) structure [126]. Furthermore, Muto and colleagues reported that Lico-A inhibits the aggregation of βA1–42, and other licochalcone derivatives also exhibit effectiveness in this process. Specifically, Lico-E demonstrates the ability to inhibit Aβ1–42 aggregation and microglial activation, promoting the activation of neuroprotective microglia M2 [56]. These findings suggest that Lico-A may hold promise as a potential drug candidate for AD treatment, owing to its ability to disaggregate βA protofibrils. Moreover, in vitro studies conducted on the SHSY5Y cell line indicate that Lico-A exerts neuroprotective effects against βA25–35-induced neurotoxicity. Specifically, Lico-A inhibited cytotoxicity caused by oxidative stress, mitochondrial dysfunction, and apoptosis [57]. The authors proposed that Lico-A modulates the PI3K/AKT/mTOR signaling pathway, with downstream suppression of mTOR-dependent autophagy potentially playing a role [127].

Additionally, Lee and colleagues synthesized new chemical compounds derived from Lico-A and coumarin (chalcone–coumarin hybrid). They investigated the compounds’ ability to prevent βA aggregation, their antioxidant properties, and radical scavenging effects in cell cultures. The study revealed that Lico-A and the synthetic compound derived from it, LM-031, exert neuroprotective effects on βA-GFP SH-SY5Y cells by activating CREB-dependent survival and upregulating the Bcl2-antiapoptotic pathway [15]. These findings suggest that Lico-A and this synthetically derived compound could be valuable in modifying the progression of AD with a multitarget approach (Figure 2).

Moreover, in vivo data reported by Lin and colleagues demonstrated that LM-031 and analogous compounds exhibit beneficial effects on learning and memory improvement in hyperglycemic 3 × Tg-AD mice [128]. This effect may be attributed to the upregulation of Nrf2 and pCREB, as well as the reduction in βA and tau levels in the hippocampus and cortex of mice.

All these data underpin that Lico-A’s ability to destabilize beta-amyloid protofibrils directly targets one of the main hallmarks of AD pathology.

### 3.6. Licochalcone A as an Acetylcholinesterase Inhibitor and Memory Enhancer

A therapeutic approach in AD involves the development of acetylcholinesterase (AChE) inhibitors based on the cholinergic hypothesis formulated by Davies and Maloney in 1976 [129]. A notable characteristic of neuronal synapses in patients with AD is diminished levels of the neurotransmitter acetylcholine (ACh) along with decreased activity of choline acetyltransferase (ChAT). ACh is released into synapses through presynaptic neuron exocytosis upon depolarization, binding to nicotinic or muscarinic receptors on postsynaptic neurons and facilitating neurotransmission [130]. AChE is the enzyme responsible for hydrolyzing ACh at synapses, converting it into choline and acetic acid, thereby deactivating cholinergic neurotransmission. A popular strategy for AD treatment involves inhibiting AChE to prevent the reduction in ACh levels in patients [131,132].

Miyazakiand colleagues reported Lico-A’s inhibitory effect on AChE activity, suggesting its potential as a drug candidate for cognitive disorder treatment [132]. Similarly, Lee and colleagues demonstrated the inhibitory effect of Lico-A on AChE activity in Tet-On Aβ-GFP 293/SH-SY5Y cells [15]. Budziak-Wieczorek and colleagues highlighted Lico-A’s neuroprotective properties, showcasing its antioxidant activity as well as its inhibition of both AChE and butyrylcholinesterase (BuChE) enzymes (AChE IC50 23.41 ± 0.02 µM, BuChE IC50 42.28 ± 0.06 µM) [14].

By preventing the degradation of acetylcholine, Lico-A’s acetylcholinesterase inhibitor activity enhances cholinergic transmission, which is impaired in AD. Hence, Lico-A has emerged as a promising candidate in neuroscience research due to its potential as an acetylcholinesterase inhibitor and memory enhancer, although more studies are necessary to confirm its effectiveness in vivo.

## 4. Current Clinical Application of Licochalcone A

As discussed in the previous section, preclinical data revealed that Lico-A exhibits multitarget effects that address several of the key mechanisms underlying AD pathogenesis, including synaptic dysfunction, neuroinflammation, oxidative stress, and amyloid accumulation. Its diverse bioactivities provide a multifaceted approach to combat the heterogeneous molecular pathology of this complex neurodegenerative disease. Further research on Lico-A promises to elucidate its therapeutic potential for AD treatment; however, unfortunately, no clinical studies involving systemic treatment of diseases with Lico-A are currently being conducted. Some key barriers to Lico-A’s clinical development include insufficient data on pharmacokinetics, bioavailability, dosing, and safety/toxicity with systemic administration. While the multi-target potential of Lico-A is promising, overcoming these challenges through rigorous preclinical research is essential to justify and enable clinical evaluation for neurological disorders like AD.

Meanwhile, clinical research on Lico-A has primarily focused on its application in the treatment of skin diseases, leading to the development of commercial preparations for topical use (Table 2). Hence, clinical trials have explored the efficacy of Lico-A for inflammatory skin conditions. Weber and colleagues reported the effectiveness of Lico-A in treating erythema [133]. Similarly, topical licorice extracts rich in Lico-A demonstrated a strong anti-irritant effect against UV-induced erythema [134]. Lico-A has also been formulated into cream preparations, along with other compounds, as an adjunctive treatment to topical retinoid therapy [135,136,137]. The study showcased a significant reduction in total lesions, fewer inflammatory lesions, and decreased skin irritations in patients treated with the moisturizer. Therefore, although the clinical application of Lico-A in dermatological conditions is promising, further clinical investigations are needed to explore its potential for systemic treatment of diseases.

## 5. Conclusions

The limited efficacy of currently available FDA-approved drugs for AD treatment, along with the absence of disease-modifying agents, has prompted extensive research, particularly among medicinal chemists, to explore novel chemical scaffolds for potential AD therapeutics.

The pathophysiology of AD is multifactorial and involves multiple molecular targets and pathways. Traditional drug discovery approaches typically focus on single targets, which may not effectively address the complexity of the disease. Multitarget compounds, on the other hand, have the potential to modulate multiple targets simultaneously, providing a more comprehensive and synergistic therapeutic approach. By targeting multiple aspects of the disease pathology, multitarget compounds can enhance therapeutic efficacy, delay disease progression, and potentially offer disease-modifying effects. Additionally, multitarget compounds may have the advantage of reducing the risk of developing drug resistance, which is a significant concern in the treatment of complex diseases. Overall, the discovery and development of multitarget compounds hold great promise in the pursuit of effective treatments for complex diseases like AD, offering new avenues for more successful therapeutic interventions. Chalcone and its analogs have shown significant potential in this regard, with numerous derivatives reported to possess multi-targeted functions.

Preclinical data have shown that Lico-A exhibits neuroprotective effects and cognitive enhancement in the context of AD. Its multifunctional mechanisms include regulating T and B cell proliferation, inhibiting βA aggregation, inhibiting AChE, and providing neuroprotection through antioxidant and anti-inflammatory effects. Furthermore, its ability to penetrate the blood–brain barrier (BBB) [138] and its potential for combination therapy makes it an exciting target for future research. Lico-A holds significant promise as a natural compound for AD treatment, potentially contributing to an improved quality of life for individuals affected by this devastating disease. While research is still in the early stages, the current evidence presented in this review supports Lico-A’s promise as a natural compound with neuroprotective potential for AD therapy. However, further preclinical and clinical studies are required to fully elucidate its therapeutic efficacy and safety profile.

## Figures and Tables

**Table 1:** Licochalcone A as a neuroprotective compound.

Row	Reference	Experimental Model	Main Findings
1	[56]	Molecular docking and Molecular dynamics simulations	Licochalcone A lead to a conformational disruption of the Aβ(1–42) protofibril.
2	[57]	SH-SY5Y cells	Licochalcone A shows a neuroprotective effect against Aβ-induced neurotoxicity, 5–35 inhibiting oxidative stress, mitochondrial dysfunction, and apoptosis. The proposed mechanism is through the activation of the PI3K/Akt/mTOR signaling pathway in SH-SY5Y cells.
3	[15]	Tet-On Aβ-GFP293/SH-SY5Y cells	Licochalcone A reduce oxidative stress, activate CREB-dependent BDNF/AKT/ERK signaling pathway involved in cell survival and CREB-dependent BCL2 for antiapoptosis.
4	[15,57,58,59]	Aβ-GFP 293/SH-SY5Y/RAW 264.6/BV-2 cells.	Licochalcone A ameliorate Aβ-induced aggregation, oxidative stress and promote neurite outgrowth in neuron like cells. Likewise, Licochalcone A prevents microglia-mediated inflammation.
5	[60]	Kainic Acid (model of temporal lobe epilepsy)	Inhibition of JNK1 by Licochalcone A can prevent neuronal degeneration in a mice experimental model of temporal lobe epilepsy TLE.
6	[61]	Rat primary cortical neurons culture	Neuroprotective properties of Licochalcone A against oxygen-glucose deprivation/reperfusion in cortical neurons could be explained trough the activation of the SIRT1/Nrf2 signaling and the inhibition of downstream NF-κB signaling pathway
7	[62]	Rat primary microglia culture	Licochalcone A exerts anti-neuroinflammatory and anti-oxidative effects in primary rat microglia mainly dependent on the arachidonic acid/COX-2/PGE2 pathway.
8	[63]	Lipopolysaccharide (LPS)-induced PD models in vivo and in vitro	Licochalcone A inhibits LPS-induced microglial activation via downregulation the activation of ERK1/2 and NF-κB p65 pathways. Licochalcone A treatment prevents dopaminergic neurodegeneration by inhibiting microglia-mediated neuroinflammation.
9	[64]	Neuropathic pain in a rat model.	Licochalcone A exerts protective effects against CCI-evoked neuropathic pain in rat model, through inhibiting microglia activation, p38 phosphorylation and inflammatory response.

**Table 2:** Licochalcone A in clinical research.

Row	Reference	Clinical Trial	Main Findings
1	[136]	NCT04002024	The study demonstrated the efficacy and safety of moisturizing cream containing licochalcone A, decanediol, L-carnitine, and salicylic acid as maintenance therapy in patients with acne of mild to moderate severity.
2	[135]	NCT02173054	The use of a moisturizer containing licochalcone A, L-carnitine, and 1,2-decanediol in addition to adapalene gel might be superior to placebo in reducing skin irritations and improving the efficacy of patient adherence to pain medications.
